# Distinct aneuploid evolution of astrocytoma and glioblastoma during recurrence

**DOI:** 10.1038/s41698-023-00453-1

**Published:** 2023-09-23

**Authors:** Jinsen Zhang, Yuan Feng, Guanghao Li, Jianhua Zhang, Xin Zhang, Yi Zhang, Zhiyong Qin, Dongxiao Zhuang, Tianming Qiu, Zhifeng Shi, Wei Zhu, Rui Zhang, Yonghe Wu, Haikun Liu, Dandan Cao, Wei Hua, Ying Mao

**Affiliations:** 1 Department of Neurosurgery, Huashan Hospital, Fudan University, Shanghai, 200040 China; 2 National Center for Neurological Disorders, Shanghai, 200040 China; 3 Shanghai Key Laboratory of Brain Function Restoration and Neural Regeneration, Shanghai, 200040 China; 4 Neurosurgical Institute of Fudan University, Shanghai, 200040 China; 5 Shanghai Clinical Medical Center of Neurosurgery, Shanghai, 200040 China; 6 Genetron Health (Beijing) Co. Ltd., Beijing, 102206 China; 7 Shanghai KR Pharmtech, Inc., Ltd, Shanghai, 201805 China; 8 Shanghai Institute for Advanced Immunochemical Studies, ShanghaiTech University, Shanghai, 201210 China; 9 Division of Molecular Neurogenetics, German Cancer Research Center (DKFZ), DKFZ-ZMBH Alliance, Im Neuenheimer Feld 280, Heidelberg, Germany

**Keywords:** CNS cancer, Cancer genomics

## Abstract

Astrocytoma and glioblastoma (GB) are reclassified subtypes of adult diffuse gliomas based on distinct isocitrate dehydrogenase (*IDH*) mutation in the fifth edition of the WHO Classification of Tumors of the Central Nervous System. The recurrence of gliomas is a common and inevitable challenge, and analyzing the distinct genomic alterations in astrocytoma and GB could provide insights into their progression. This study conducted a longitudinal investigation, utilizing whole-exome sequencing, on 65 paired primary/recurrent gliomas. It examined chromosome arm aneuploidies, copy number variations (CNVs) of cancer-related genes and pathway enrichments during the relapse. The veracity of these findings was verified through the integration of our data with multiple public resources and by corroborative immunohistochemistry (IHC). The results revealed a greater prevalence of aneuploidy changes and acquired CNVs in recurrent lower grade astrocytoma than in relapsed grade 4 astrocytoma and GB. Larger aneuploidy changes were predictive of an unfavorable prognosis in lower grade astrocytoma (*P* < 0.05). Further, patients with acquired gains of 1q, 6p or loss of 13q at recurrence had a shorter overall survival in lower grade astrocytoma (*P* < 0.05); however, these prognostic effects were confined in grade 4 astrocytoma and GB. Moreover, acquired gains of 12 genes (including *VEGFA*) on 6p during relapse were associated with unfavorable prognosis for lower grade astrocytoma patients. Notably, elevated *VEGFA* expression during recurrence corresponded to poorer survival, validated through IHC and CGGA data. To summarize, these findings offer valuable insights into the progression of gliomas and have implications for guiding therapeutic approaches during recurrence.

## Introduction

Astrocytoma and glioblastoma (GB) have undergone redefinition as distinct subtypes of adult diffuse gliomas, as delineated in the fifth edition of the WHO Classification of Tumors of the Central Nervous System (WHO CNS5)^1^. Notably, the inevitability of recurrence amplifies the challenge, underscoring the urgency for interventions^2^. To unravel the intricate differences of astrocytoma and GB progression, characterized by disparate metabolic pathways, evolutionary trajectories, and treatment response^3–5^, a comprehensive grasp of the dynamic molecular events is indispensable. Longitudinal analysis of glioma samples has unveiled a spectrum of evolutionary mechanisms including driver gene mutations, copy number variations (CNVs), and chromosome aneuploidy^6–8^. However, achieving consensus on this intricate matter remains an ongoing pursuit in this field.

Through genomic analysis of paired primary and recurrent tumors^6,9–13^, several canonical driver genes and related oncogenic pathways promoting glioma progression and deciphering the evolutionary trajectory have been delineated. Notably, investigations have shown that for grade 2/3 (lower grade) gliomas, the deletion of *CDKN2A/B* was observed in 61% of patients, *PTEN* in 39%, and amplification of *MYC* in 22%, as well as *FOXM1* in 20% during progression^10^. In contrast, *EGFR* amplification as maintained in 34% of recurrent tumors and 42% of primary tumors, and *TERT* promoter mutation was observed in 100% of recurrent tumors and closely 98% of primary tumors in GB patients^11,12^. These findings indicate distinct genetic alterations that differentiate the evolution paths of astrocytoma and GB.

Beyond gene alterations, aneuploidy characterized by an irregular count of chromosomes stands out as a recurrent feature in gliomas and the recurrence process^14^. Within the realm of gliomas, a consensus has crystallized around aneuploidy, signifying 1p/19q codeletion as a pivotal clonal event and a diagnostic hallmark exclusive to oligodendroglioma. Conversely, in the context of GB patients, the concurrent gain of chromosome 7 and loss of chromosome 10 (+7/−10) serve as indicator of unfavorable prognosis^15^. Notably, *IDH*-mutant and 1p/19q-intact glioma exhibit heightened instances of acquired aneuploidy^16^. It is important to recognize that aneuploidy plays divergent roles contingent on genetic context and tumor type, emphasizing its context-specific and tumor-subtype-dependent functionality^17,18^. Despite this, the comprehensive implications of aneuploidy and its interaction with pivotal genes during the recurrence of astrocytoma and GB remain incompletely elucidated, especially in the context of WHO CNS5 segregating them into two distinct categories.

In this study, we collect a cohort of 65 paired primary and recurrent gliomas and conduct whole-exome sequencing. Through analyses of aneuploidy and CNVs in astrocytoma and GB, we reveal differences in the features of aneuploidy and aneuploidy-associated gene alterations, as well as their prognostic value in the recurrence process, across lower grade astrocytoma, grade 4 astrocytoma, and GB.

## Results

### Characteristics of Paired Primary and Recurrent Glioma from 65 Patients

**Fig. 1:**
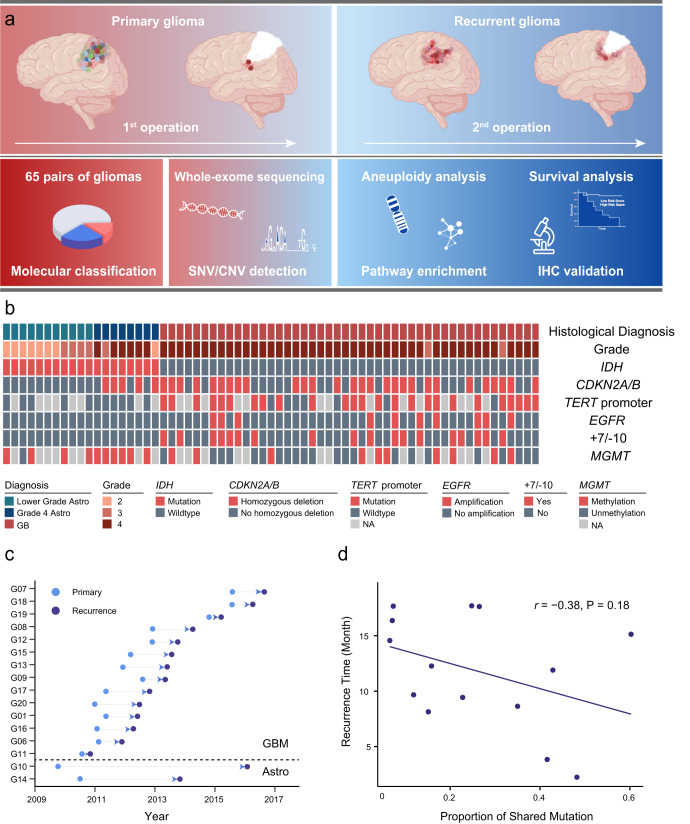
Study workflow and characteristics of paired primary and recurrent glioma. **a** Sixty-five paired primary and recurrent glioma samples including grade 2/3 (lower grade) astrocytoma (*n* = 11), grade 4 astrocytoma (*n* = 8) and GB (*n* = 46) were enrolled to perform the whole-exome sequencing. Copy number variations (CNVs) and single nucleotide variants (SNVs) were detected for further evaluating of aneuploidy, pathway enrichment. Survival analysis and immunohistochemistry (IHC) validation were also performed as depicted (Created with BioRender.com). **b** Diagnosis, pathological, and molecular characteristics of primary glioma patients. Each column represents a patient. **c** Surgery time of primary and recurrent glioma patients. Sixteen cases were classified into 14 astrocytoma (Astro) and 2 glioblastoma (GB) patients. **d** The correlation between the proportion of shared mutations and recurrence time in GB. *r* denotes Pearson correlation coefficient. P values were obtained from the F test.

Sixty-five patients with both paired primary and recurrent post-surgery samples were included in the current study (Fig. 1a). All tumors were localized within the cerebral hemispheres, with the majority situated in the frontal (35 cases) and temporal (22 cases) lobe (Supplementary Fig. 1a). According to the classification and grading guidelines delineated by WHO CNS5, the 65 glioma patients were stratified into three distinct categories: grade 2/3 (lower grade) astrocytoma (*n* = 11), grade 4 astrocytoma (*n* = 8), and GB (*n* = 46) (Supplementary Fig. 1b). Of these *IDH*-mutant astrocytoma cases, two characterized by lower-grade histologic features were reclassified as grade 4 astrocytoma due to the presence of *CDKN2A/B* homozygous deletion, while six displayed grade 4 histology (Fig. 1b). The representation of characteristic molecular markers, including *IDH* mutation, 1p/19q codeletion, *TERT* promoter mutation, *MGMT* methylation and *BRAF* V600E mutation, were also elucidated in Fig. 1b. Further clinical and molecular characteristics, along with recurrence-free intervals, were summarized in Dataset 1 and Supplementary Fig. 1.

Whole-exome sequencing was performed on 65 pairs of primary and recurrent gliomas, achieving a median coverage of 490x (Dataset 2). Among these, 16 cases were accompanied by corresponding blood samples, and single-nucleotide variants (SNVs) were identified (Dataset 3, 4). The mutations within pivotal driver genes, such as *TP53*, *EGFR*, *NF1*, and *PIK3CA*, exhibited frequent occurrence in the context of GB (Supplementary Fig. 2). The proportion of shared SNVs observed in paired primary and recurrent GB cases (26.4%, *n* = 14) was higher than that of astrocytomas (15.8%, *n* = 2), and GB had a much shorter recurrence time compared to astrocytomas (12 *vs* 57 months, Fig. 1c). Additionally, an inverse correlation was observed between the proportion of shared SNVs among primary and recurrent GB and the recurrence-free interval time (r = −0.38, *P* = 0.18, F test, Fig. 1d). The genetic association between primary and recurrent astrocytoma was found less intimate than that in GB, a characteristic that might contribute to the longer interval to relapse in astrocytoma cases.

### Higher aneuploidy difference during relapse of lower grade astrocytoma compared to grade 4 astrocytoma and GB

**Fig. 2:**
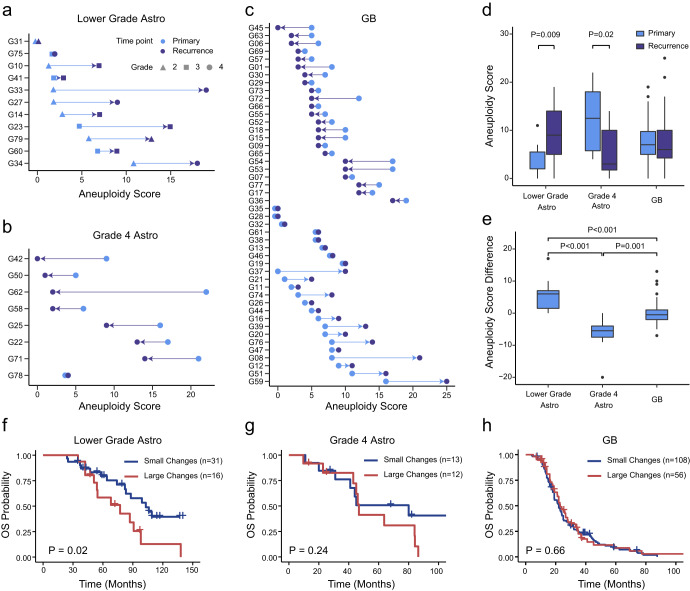
Aneuploidy changes during relapse of lower grade astrocytoma, grade 4 astrocytoma and GB. **a** Aneuploidy score for lower grade astrocytoma (*n* = 11) and their recurrence. **b** Aneuploidy score for grade 4 astrocytoma (*n* = 8) and their recurrence. **c** Aneuploidy score for GB (*n* = 46) and their recurrence. Points in light blue color represent primary samples, and in dark blue represent recurrent samples. **d** Boxplot of aneuploidy score for the primary and recurrent lower grade astrocytoma, grade 4 astrocytoma and GB. **e** Aneuploidy score difference (recurrent - primary) between primary and recurrent samples for lower grade astrocytoma, grade 4 astrocytoma and GB. For boxplots in (**d**) and (**e**), the upper and lower boundaries represent the first and third quantiles, respectively. The horizontal lines represent the median, and the whiskers denote the lowest and highest values except for outliers (black dots). Overall survival for primary lower grade astrocytoma (**f**), grade 4 astrocytoma (**g**) and GB patients (**h**) with small or large aneuploidy changes. Survival analysis was performed with the combination of our data (*n* = 49) and data downloaded from cBioPortal (*n* = 187). Cases with aneuploidy difference bigger than 3 or smaller than −3 was classified into large change group, others were classified into small change group. *P* values were obtained from the log-rank test.

Aneuploidy has been identified as a prognostic indicator for primary *IDH*-mutant astrocytic gliomas^19^. In this study, we investigated the changes in aneuploidy levels during the transition from primary to recurrent stages in three distinct glioma groups. The aneuploidy score for each sample was calculated through counting the number of amplified and deleted chromosome arms (Materials and Methods, Dataset 5). We observed that the divergent patterns of aneuploidy changes during relapse across lower grade astrocytoma, grade 4 astrocytoma, and GB. For lower grade astrocytoma, the recurrent tumors manifested a notably higher aneuploidy score (Fig. 2d, e, Wilcoxon rank-sum test, *P* = 0.009), and, for almost all cases, aneuploidy increased (Fig. 2a). On the contrary, the dynamics of aneuploidy alternations during the relapse of GBs exhibited heterogeneity. Here, the aneuploidy score increased in 15 cases, remained unchanged in 8 cases, and decreased in 23 cases (Fig. 2c), and the recurrent GBs exhibited no statistically significant deviation from their primary counterparts (Wilcoxon rank-sum test, *P* = 0.60, Fig. 2d, e), which was consistent with previous research^16^. Notably, the changes in aneuploidy for grade 4 astrocytoma were quite different. The recurrent tumors displayed a significantly lower aneuploidy (Wilcoxon rank-sum test, *P* = 0.02, Fig. 2d, e), and nearly all cases exhibited a decrease in aneuploidy at recurrence (Fig. 2b). Consequently, it is noteworthy that aneuploidy generally exhibited an escalating trend correlated with higher grade during the recurrence of lower grade astrocytoma. Conversely, for grade 4 glioma, the occurrence of increased aneuploidy during recurrence was not frequently observed.

The relationship between aneuploidy and prognosis was further investigated using both our data and public data from cBioPortal^20,21^. Notably, patients with lower grade astrocytoma who experienced large changes in aneuploidy levels during relapse had significantly shorter overall survival (log-rank test, *P* = 0.02, Fig. 2f), corroborating previous findings^16^. However, no significant effects were observed for grade 4 astrocytoma and GB patients (log-rank test, *P* = 0.24 for grade 4 astrocytoma, *P* = 0.66 for GB, Fig. 2g, h).

### More chromosome arm aneuploidies were acquired during relapse of lower grade astrocytoma than grade 4 astrocytoma and GB

**Fig. 3:**
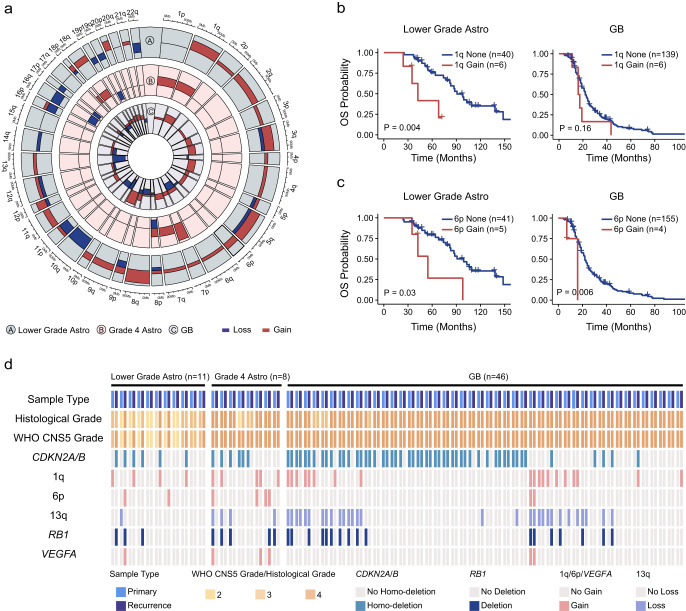
Chromosome arm aneuploidy acquired and their effects on prognosis during the relapse of lower grade astrocytoma, grade 4 astrocytoma and GB. **a** Newly acquired chromosome arm aneuploidy during the relapse of lower grade astrocytoma (outer-most circle, *n* = 11), grade 4 astrocytoma (middle circle, *n* = 8) and GB (inner-most circle, *n* = 46). Copy number variations of 39 chromosome arms were showed. Red color represents gain and blue represents loss. Patients in this analysis were all from this study. Overall survival for lower grade astrocytoma and GB patients with or without newly acquired 1q gain (**b**) and 6p gain (**c**) at recurrence. *P* values were obtained from the log-rank test. Survival analysis was performed with the combination of our data (*n* = 49) and public data downloaded from cBioPortal (*n* = 187). Cases without arm gain (loss) in both primary and recurrent tumors were classified into “None” group. Cases with arm gain (loss) in recurrent tumor and without gain (loss) in primary tumor were classified into “Gain (Loss)” group. **d** CNVs in arm levels (1q, 6p and 13q) and gene levels (*RB1*, *VEGFA*) in primary and recurrent tumors for 65 patients. Histological grade and WHO CNS5 grade were shown.

We subsequently compared CNVs of recurrent tumors with their corresponding primary counterparts using our data in order to identify the chromosome arm aneuploidy that propelled the progression of gliomas. Our findings indicated a frequent presence of arm aneuploidy, with CNVs acquired during malignant evolution being distributed extensively nearly all chromosomes (Fig. 3a). Overall, in lower grade astrocytoma, on average, 16.4% of arm-level alterations identified in primary tumors were no longer detectable at recurrence, 17.2% were detected in both primary and recurrent tumor, and 66.4% were only detected at recurrence. However, for grade 4 astrocytoma, the three values were 59.8, 29.5 and 10.7%, respectively. For GB, the three values were 22.6, 54.5 and 22.9%, respectively. Thus, the proportion of arm aneuploidy acquired during the progression of lower grade astrocytoma was much higher than that of grade 4 astrocytoma and GB.

We also investigated the impact of newly acquired arm aneuploidy on patient prognosis by utilizing both our data and public data^20,21^. Our analysis illuminated that lower grade astrocytoma patients who acquired the gain of 1q, 6p or the loss of 13q upon recurrence had significantly shorter overall survival in contrast to those without these alterations (log-rank test, *P* = 0.004 for 1q gain, *P* = 0.03 for 6p gain, *P* = 0.04 for 13q loss, Fig. 3b, c, Supplementary Fig. 3). Notably, the emergence of 1q gain, 6p gain, and 13q loss during recurrence was associated with progression for lower grade astrocytoma. In our data, three cases acquired 1q gain during recurrence showed progression (two grade 2 to 4, one grade 3 to 4, Dataset 6) considering *CDKN2A/B* homozygous deletion. Similarly, two cases with 6p gain advanced from grade 2 to 4 (Fig. 3d, Dataset 7). Loss of 13q was identified only in primary tumor of one case in our data, while in public data, six cases acquired 13q loss showed progression (Dataset 8). In contrast, for GB, only the gain of 6p was an indicator of unfavorable prognosis (log-rank test, *P* = 0.006, Fig. 3c). But this arm aneuploidy was detected only in 2.5% (4/159) of recurrent GB cases. Consequently, the acquired chromosome arm aneuploidy was associated with a worse prognosis in lower grade astrocytoma. On the contrary, in grade 4 astrocytoma and GB, the acquired arm-level alterations had a limited effect on prognosis.

### More cancer-related genes and pathways altered during the relapse of lower grade astrocytoma than grade 4 astrocytoma and GB

**Fig. 4:**
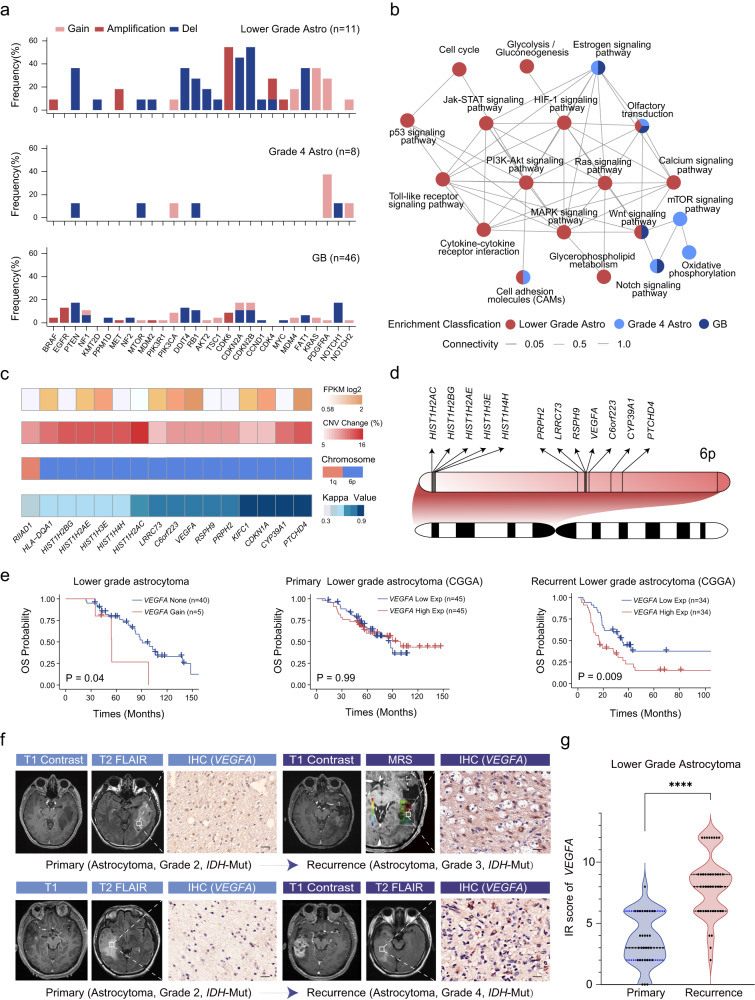
Candidate driver genes and cancer-related pathways during the relapse of lower grade astrocytoma, grade 4 astrocytoma and GB. **a** Newly acquired canonical driver gene alterations during the relapse of lower grade astrocytoma, grade 4 astrocytoma and GB. Horizontal axis represents driver gene, and vertical axis represents the proportion of alterations in all samples. Dark red color represents amplification, light red represents gain, and blue represents deletion. **b** Enrichment network of cancer related KEGG pathways during the relapse of lower grade astrocytoma, grade 4 astrocytoma and GB. Each circle represents one pathway. Circles in red, light blue and dark blue represent they were enriched in lower grade astrocytoma, grade 4 astrocytoma and GB, respectively. Circles with multiple colors represents they were enriched in multiple groups. **c** Profiling of 16 genes with consistent transcriptional and CNV changes in 1q and 6p. Expression profile, CNV alteration frequency, chromosome arm status, and kappa value between chromosome arm and gene alteration were showed from the top to the bottom panel. Expression data was download from CGGA database and public CNV data was from cBioPortal database. Orange color represents up-regulated genes on the top of the panel. Red color represents gain on the middle of the panel. **d** Location of the 12 prognosis-related genes in 6p. The genomic coordinate increase from left to the right of the panel. Line of each gene represents its position in the chromosome arm. **e** Overall survival for lower grade astrocytoma patients with or without *VEGFA* gain (5 patients from this study, 40 patients from cBioPortal), recurrent lower grade astrocytoma patients with high expression of *VEGFA* and low expression of *VEGFA*, primary lower grade astrocytoma patients with high expression of *VEGFA* and low expression of *VEGFA*. *P* values were obtained from the log-rank test. **f** IHC staining of *VEGFA* and MRI images on paired primary and recurrent glioma. Patient 1 was initially diagnosed with a lower grade astrocytoma (*IDH*-mutant, grade 2 astrocytoma) in 2009 with T1 and T2 FLAIR imaging showing a temporal lesion. In 2016, the tumor recurred as a grade 3 astrocytoma (*IDH*-mutant), and T1 contrast imaging revealed tumor enhancement and a high Cho/NAA ratio. Patient 2 was initially diagnosed with a lower grade astrocytoma (*IDH*-mutant, grade 2 astrocytoma) in 2010 with T1 and T2 FLAIR imaging showing a temporal lesion. In 2012, the tumor recurred as a grade 4 astrocytoma (*IDH*-mutant), and T1 contrast imaging revealed tumor enhancement. **g** The mean immunoreactivity score (IRS score) of *VEGFA* was compared between primary and recurrent gliomas. P values was obtained from the Wilcoxon rank-sum test.

We also observed that more canonical driver genes were acquired during the recurrence of lower grade astrocytoma compared to grade 4 astrocytoma and GB. Specifically, in over 30% of patients with lower grade astrocytoma, new deletion of *PTEN*, *CDKN2A*, *CDKN2B*, or amplification of *CDK6* and gain of *KRAS* were detected (Fig. 4a), which is consistent with previous reports^10^. In contrast, in grade 4 astrocytoma, only gain of *PDGFRA* was acquired in 3 of 8 patients, while the remaining 6 genes were newly altered in only one patient (Fig. 4a). Similarly, in GB, the incidence of newly acquired alterations in driver genes were infrequent, with only deletion of *PTEN*, *NOTCH1*, *DDIT4* and amplification of *EGFR* being more than 10% (Fig. 4a, Dataset 9).

To uncover the oncogenic pathways driving glioma progression, we performed KEGG enrichment analysis using genes with high frequency CNVs acquired during the relapse (Materials and Methods, Dataset 10, 11, 12). Specifically, lower grade astrocytoma was found to share the cell adhesion molecules pathway with grade 4 astrocytoma, and the Wnt signaling pathway with GB, while Notch and estrogen signaling pathways were enriched in both grade 4 astrocytoma and GB. We also identified a shared enriched pathway among all three glioma groups, that is the olfactory transduction pathway (Fig. 4b). Moreover, there were many more cancer-related pathways enriched during the relapse of lower grade astrocytoma compared to grade 4 astrocytoma and GB, such as HIF-1, PI3K-Akt, Jak-STAT signaling pathways (Fig. 4b). These results suggested that the recurrent process of lower grade astrocytoma required more alterations within tumorigenic pathways.

### Genes located on 6p were associated with the prognosis of lower grade astrocytoma

We further explored potential driver genes by analyzing the expression and CNVs of genes residing on three chromosome arms (1q, 6p and 13q), which had demonstrated a significant relevance to prognosis. We identified 16 genes that consistently showed transcriptional upregulation concurrent with copy number gains, all of which belonged to lower grade astrocytoma (Materials and Methods, Fig. 4c, Dataset 13). We observed a high concordance between gene alterations and their chromosomal arms, with 93.8% (15/16) of these genes demonstrating at least a moderate degree of concordance (kappa > 0.4) with the respective arms they were located on (Fig. 4c). Additionally, survival analysis showed that among these genes, 12 genes situated on 6p were associated with poor prognosis in lower grade astrocytoma (Dataset 13). Remarkably, the majority of these 12 genes were densely clustered within several large genomic segments on 6p (Fig. 4d), with, for example, five histone-coding genes being located adjacently. These results suggested that these clusters of genes may function together with the chromosome arm to promote the recurrence of lower grade astrocytoma.

### *VEGFA* was a potential prognostic marker for the recurrence of lower grade astrocytoma

*VEGFA*, a key mediator of angiogenesis in cancer, was found to be one of the 12 prognosis-related genes. In the case of lower grade astrocytoma, the acquisition of *VEGFA* gain upon recurrence was found to be significantly correlated with shorter overall survival (Fig. 4e, log-rank test, *P* = 0.04). By analyzing the transcriptional profiling of glioma from CCGA (Materials and Methods)^22–24^, it revealed that higher expression of *VEGFA* was also associated with poor prognosis for recurrent lower grade astrocytoma (Fig. 4e, log-rank test, *P* = 0.009). However, this association wasn’t replicated in primary lower grade astrocytoma (Fig. 4e, log-rank test, *P* = 0.99), primary and recurrent grade 4 astrocytoma, and GB (Supplementary Fig. 4). The expression of *VEGFA* was further validated with IHC staining in other independent paired samples. The mean immunoreactivity score (IR score) of the recurrent lesions was significantly higher than that of their primary counterparts (Fig. 4f, g, Wilcoxon rank-sum test, *P* < 0.0001). Collectively, these results indicated that *VEGFA* was a prognostic marker specifically within the context of recurrent lower grade astrocytoma.

## Discussion

Glioma recurrence is a critical factor associated with poor prognosis, and investigating the recurrent process and underlying mechanism is imperative. Here we report a single-center longitudinal genomic analysis encompassing 65 paired primary and recurrent glioma samples. Our findings show a distinct pattern wherein a higher prevalence of chromosome arm aneuploidy and single-gene-locus CNVs are evident during the relapse of lower grade astrocytoma, in contrast to grade 4 astrocytoma and GB. Additionally, patients with larger aneuploidy changes or acquired gain of 1q, 6p or loss of 13q have significantly shorter overall survival than those without these alterations for lower grade astrocytoma. Specially, the presence of *VEGFA* gain indicates an unfavorable prognosis in recurrent lower grade astrocytoma patients.

Aneuploidy can exhibit contrasting implications across diverse cancer types. For instance, it might align with a favorable prognosis in conditions like multiple myeloma and acute lymphoblastic lymphoma, both characterized by a heightened degree of aneuploidy^25,26^, while it conversely associates with an unfavorable prognosis in breast cancer, colorectal cancer, and prostate cancer^17,27,28^. Since the function of aneuploidy is tumor-type dependent, specific changes of aneuploidy and its prognostic implications during astrocytoma and GB relapse have seldom been reported. In our study, we found that more aneuploidy accumulates during the recurrence of lower grade astrocytoma compared to grade 4 astrocytoma and GB. Furthermore, we demonstrated that aneuploidy acquired during the course of malignant progression could serve as an independent prognostic marker. Consistent with the results of the GLASS Consortium study, patients with *IDH*-mutant and 1p/19q-intact glioma acquired higher level of aneuploidies at recurrence and carried more cell cycle gene alterations related to poor survival in our cohort^16^. For lower grade astrocytoma, characterized initially by fewer genetic alterations, it is reasonable to expect high levels of aneuploidy to accumulate during a long progression-free time to drive tumor recurrence. This may be due to the maintained genetic alternations that are inherently present in GB, such as *EGFR* amplification, *TERT* promoter mutation, *PTEN* and *NF1* mutation, which can drive GB recurrence in a short time.

In our study, we investigated genes located on chromosome arm 6p that are associated with prognosis in astrocytoma, and identified 16 genes that showed a high concordance between transcription expression and chromosome arm CNVs. Specifically, we found that gain of *VEGFA* in lower grade astrocytoma patients had significant prognostic value and the expression of *VEGFA* was further validated to be upgraded at recurrence by IHC. During glioma progression, hypoxia and necrosis develop centrally and present as the features of high-grade glioma. In this intricate interplay, it is known that *VEGFA* expression is upgraded via the activated MAPK (ERK) pathway in hypoxic conditions^29^. In this study, we identified the high expression of *VEGFA* in necrosis regions of high-grade gliomas (Supplementary Fig. 5). However, clinical trials targeting *VEGFA* in gliomas have not achieved the anticipated goals, as the administration of bevacizumab, an anti-VEGFA drug approved by the FDA for recurrent GB, has shown limited survival benefit^30^. Such interventions could potentially activate the HIF-1 and MET pathways, consequently fostering epithelial-mesenchymal transition and chemo-resistance in gliomas^31–34^. Our analysis suggests that *VEGFA* gain is not the only driver event in glioma recurrence, and targeting *VEGFA* alone may not fully reverse the effect of 6p gain in aneuploid cells. Given the potential contribution of aneuploidy in glioma recurrence, combination therapy with bevacizumab may merit further investigation. It is important to note that while bevacizumab has been approved by the FDA for recurrent GB, its survival benefits are limited.

Different from other researches^16,35,36^, our study focused exclusively on astrocytoma and GB, taking into consideration the distinct genetic backgrounds of astrocytic and oligodendritic cells. As CNVs were more frequent in recurrent gliomas and indicated worse outcomes, particularly in the *IDH*-mutant subtypes of adult astrocytoma^37^, we also discovered that acquired CNVs during relapse were more common in lower grade astrocytoma than in grade 4 astrocytoma and GB. In our study, CNV profiles of 14 GB cases with matched control were analyzed, and we identified one (G06) with decrease in aneuploidy partially attributed to copy neutral loss of heterozygosity (LOH) (Supplementary Fig. 6). Additionally, the number of recurrent private SNVs was higher in lower grade astrocytoma than in primary privates, while in GB, the number of recurrent private SNVs was similar to primary private SNVs. Consequently, it appears that the genome in GB is more stable than in astrocytoma during progression. These findings are consistent with the EORTC 1542 study, which examined 186 paired glioblastomas and found a median retention rate of 80% genetic mutation at recurrence^38^. Moreover, the treatment modality for glioma can instigate genomic alterations within the tumor. For instance, radiotherapy has been shown to give rise to genomic variants, including deletions in relapsed tumors. This phenomenon subsequently triggers the activation of DNA repair pathways, which, in turn, is associated with an unfavorable prognosis^39^. Additionally, TMZ administration has been linked to a hypermutation phenotype in relapsed patients, leading to modifications within the tumor microenvironment^36^. In our study, we observed one case (G20) with hypermutation at recurrence, following radiotherapy and TMZ chemotherapy, and the aneuploidy score increased slightly from 7 to 10. And the effect of treatment on aneuploidy clearly deserves further study.

Despite several new findings regarding aneuploidy changes during the relapse of astrocytoma and GB in our study, there are still some limitations. Only 16 patients had blood samples available for analysis, which hindered comprehensive profiling of glioma genomes. Furthermore, further investigation is needed to determine the effect of radio-chemotherapy, target therapy, and tumor-treating fields on molecular alterations. As gliomagenesis can be regulated at multiple levels, integrated multi-omics analysis is needed to identify new therapies.

## Methods

### Sample collection and DNA extraction

Formalin-fixed and paraffin-embedded (FFPE) tumor samples, as well as matched blood samples, were obtained from 65 patients with diffuse gliomas at the Department of Neurosurgery, Huashan Hospital of Fudan University from 2000 to 2018. The study was approved by the institutional review board of Huashan Hospital, Fudan University, and written informed consent was obtained from all patients (receiving ethics committee number KY2022-496). Pathological diagnoses were confirmed by two pathologists. Genomic DNA was extracted using the QIAamp DNA Mini Kit (QIAGEN, Cat#51306) following the manufacturer’s protocol. Molecular markers, including *IDH* mutation, O6-alkylguanine DNA alkyl transferase (*MGMT*) promoter methylation, chromosome 1p/19q codeletion, telomerase reverse transcriptase (*TERT*) promoter mutation, and *BRAF* V600E mutation, were detected for molecular pathology diagnosis.

### Whole-exome sequencing

Whole exome capture was performed using SureSelect Human All Exon V6 (Agilent Technologies). In brief, about 100 ng genomic DNA from each of the 65 paired tumor samples and 16 matched blood samples were extracted and fragmented using Covaris M220 (150 bp to 200 bp fragment size). The fragmented DNA was end-repaired and extended with an ‘A’ nucleotide at the 3’end. After PCR amplification and purification, the libraries were constructed using Agilent SureSelect kit according to the manufacturer’s instructions. The qualified exome-captured libraries were sequenced using Illumina HiSeq X Ten at Shanghai KR Pharmtech, Inc., Ltd. Adapters and low-quality reads were removed using trimmomatic (version 0.33)^40^. Then the clean reads were aligned to the human reference genome (hg19) with Burrows-Wheeler Aligner (BWA; version 0.7.10)^41^. All aligned reads were sorted, de-duplicated using Picard (version 1.103) (https://broadinstitute.github.io/picard/). Alignment quality assessment, local realignment and base quality recalibration were performed using the Genome Analysis Toolkit (GATK; version 3.1)^42^. Mapping rate, coverage rate and duplicate rate were calculated with qualimap (version .2.1)^43^. Soft-clipped reads were removed to reduce the influence of DNA degradation of FFPE samples.

### Detection of copy number variations (CNVs) and calculation of aneuploidy score

For 65 paired tumor samples, genomic segments were identified using CNVkit (version 0.9.9)^44^. The authors of CNVkit recommended combining all normal samples into a pooled reference, which performed slightly better than matched control as reference. According to this, we constructed a pooled reference with all blood samples (*n* = 16) as the control for CNV calling for all samples. CNVs in chromosome arm level were detected using the proportion of gain or loss regions for each chromosome arm. Briefly, we judged the status of a segment according to whether its log2 ratio is bigger than 0.170 (gain) or smaller (loss) than −0.193. Then the proportion of gain, *p*, was the total length of segments with gain divided by the whole arm length. The proportion of loss, *q*, was calculated in the same way. Finally, the chromosome arm CNV status was regarded as amplified if *p* was bigger than 0.7, deleted if *q* was bigger than 0.7, or neutral if both *p* and *q* were smaller than 0.7^16^. To detect CNVs in gene level accurately, we employed a higher cutoff. One gene was gained if its log2 ratio was bigger than 0.322 and deleted if its log2 ratio was smaller than −0.415. Specifically, one gene was amplified if its log2 ratio was bigger than 0.807, in which copy number is bigger than 5 in consideration of tumor purity (Dataset 9)^45^. Thresholds used here to identify CNVs are moderate, and we take tumor purity into account. This method is similar to the previous approaches^15,16,46–48^.

For each sample, aneuploidy score was the number of amplified and deleted chromosome arms. Aneuploidy score was calculated to investigate the progress of gliomas from primary to recurrence at chromosomal level. The range of aneuploidy score is from 0 to 39, as sex chromosomes and short arms of chromosomes 13, 14, 15, 21 and 22 were excluded.

### Detection of somatic single nucleotide variants (SNVs)

Somatic mutation calling was performed using the Mutect (version 1.1.4)^49^ for 16 paired primary and recurrent glioma samples with matched normal DNA samples. Variants were filtered based on allelic and overall depths of coverage, strand bias, and mapping quality. In brief, variants listed in dbSNP (version 141)^50^ were excluded. Variants with coverage bigger than 10, the number of variant reads more than 4 and mutant allele frequency bigger than 5% were retained. Formalin fixation introduces a bias in the type of nucleotide substitutions^51^, so we filtered the SNVs where the single-strand bias of C to T mutations is greater than 0.2^52^. Detected variants were annotated on the basis of Ensembl annotations (version 92) using Variant Effect Predictor (VEP; version 2.7)^53^. Finally, variants classified into missense mutation, nonsense mutation or splice site were retained. Oncogene were annotated using IntOGgen (https://www.intogen.org/)^54^.

### Pathways analysis for genes with CNVs in lower grade astrocytoma, grade 4 astrocytoma and GB recurrence

All samples were classified into three groups: lower grade astrocytoma (*n* = 11), grade 4 astrocytoma (*n* = 8) and GB (*n* = 46), and pathway analysis was performed for each group, separately. For each group, genes were selected for the analysis if they were amplified or deleted more than 15% samples in the recurrence, and were copy-neutral in the paired primary samples. The cutoff value (15%) was determined in consideration of different number of samples for the three groups.

KEGG (Kyoto Encyclopedia of Genes and Genomes) pathways of the selected genes were enriched by KOBAS3.0 (http://kobas.cbi.pku.edu.cn/)^55^. *P* value was adjusted by Benjamini-Hochberg method and pathways with adjusted *q* < 0.05 were selected.

### Analysis of public genomic and transcriptomic data of lower grade astrocytoma, grade 4 astrocytoma and GB

To validate our findings in more samples and perform survival analysis for CNVs at chromosome and gene levels, we download the genomic data (log ratio) of primary and recurrent glioma from cBioPortal database (https://www.cbioportal.org/), including datasets from GLASS Consortium, TCGA and MSKCC^20,21^. Cases with paired primary and recurrent tumors, and the primary glioma diagnosed with astrocytoma and GB were involved. Patients younger than 18 and older than 80 or without CNV data were excluded. In total, 62 paired astrocytoma and 138 paired GB patients were selected for subsequent analysis. We called CNVs in arm and focal levels using the same method as our data. And overall survival data was available for 187 patients (42 cases of lower grade astrocytoma, 19 cases of grade 4 astrocytoma, 126 cases of GB).

To explore the expression profiles for genes with copy number changes, we downloaded RNA-Sequencing data (mRNAseq_693) from CGGA database (http://cgga.org.cn/index.jsp)^22–24^.Cases diagnosed with astrocytoma and GB were involved. Patients younger than 18 and older than 80 were filtered out. Wilcoxon rank-sum test was used to identified differentially expressed genes (DEGs) between primary and recurrent samples with the threshold of fold-change >1.5 and adjust *p*-value < 0.05^56^.

To identify potential driver genes during the recurrence of lower grade astrocytoma, grade 4 astrocytoma and GB, we analyzed gene expression (described above), CNV, and chromosome arm aneuploidy for the three groups of gliomas separately. Genes with consistent transcriptional upregulation with amplified CNVs (more than 5% samples in our data and public data) on the amplified prognostic-related arms 1q and 6p were selected. Genes with consistent downregulation and deleted CNVs (more than 5% samples in our data and public data) on the deleted prognostic-related arms 13q were selected. We further explored the effects of these genes on prognosis through survival analysis.

### Immunohistochemistry

IHC staining was performed as described previously^57^ in the tissue samples from 13 paired lower grade astrocytoma and 8 paired GB. VEGFA Polyclonal antibody (dilution 1:200, Proteintech 19003-1-AP) was purchased from Proteintech (Wuhan, China). Three independent regions of interest (ROIs) were chosen per sample. Immunoreactivity score (IRS) was calculated to evaluate *VEGFA* expression levels by combining staining intensity with the percentage of positive cells. Staining intensity was classified as 1 of 4 grades using a scale of 0 (negative), 1 (weak), 2 (moderate), and 3 (strong). Percentage of positive cells was regarded as 0 (none), 1 (<10%), 2 (10–50%), 3 (51–80%), and 4 (>80%). The IRS (0–12) was then calculated by multiplying the score values.

### Statistical analysis

Survival curves were estimated by the Kaplan-Meier method and log-rank test was used to assess the statistical significance between different groups. Survival analysis was performed with the combination of our data and public data downloaded from cBioPortal with long-term follow-up and patients lost to follow-up immediately after the operation were excluded. To explore the association of CNV in arm and focal levels with tumor relapse, we compared overall survival of cases newly acquired alterations with those not acquired during relapse. For example, patients with 1q gain at recurrence and without 1q gain in the primary tumor were classified into “1q Gain” group. Patients without 1q gain in both primary and recurrent tumors were classified into “1q None” group. Patients with 1q gain in both primary and recurrent tumors, or only in primary tumors were not included in this analysis. Other survival analysis, including 6p, 13q, *VEGFA*, *RB1* were performed in the same way. Comparisons between the age, time to secondary surgery, aneuploidy score and IRS score of two groups were made with Wilcoxon rank-sum test to assess statistical significance. Kappa values were used to assess the agreement between the alteration of genes and arms.

### Supplementary information

Supplementary Information

Reporting-summary

Dataset

## Data Availability

The raw sequence data reported in this paper have been deposited in the Genome Sequence Archive^58^ in National Genomics Data Center^59^, China National Center for Bioinformation / Beijing Institute of Genomics, Chinese Academy of Sciences (GSA-Human: HRA004929) that are publicly accessible at https://ngdc.cncb.ac.cn/gsa-human. Public CNV profiles were downloaded from cBioPortal (https://www.cbioportal.org/). Public RNA-sequencing data (mRNAseq_693) was from CGGA (http://cgga.org.cn/index.jsp).

## Code availability

Analysis code used in this paper are available upon request to the corresponding authors.
