# Supplementary material for: Distinct aneuploid evolution of astrocytoma and glioblastoma during recurrence
Source: NPJ Precis Oncol. 2023 Sep 23;7:97. doi: 10.1038/s41698-023-00453-1 (PMC10517995; doi:10.1038/s41698-023-00453-1)
Supplement: Supplementary file 1 — Supplementary Information [file 41698_2023_453_MOESM1_ESM.pdf]

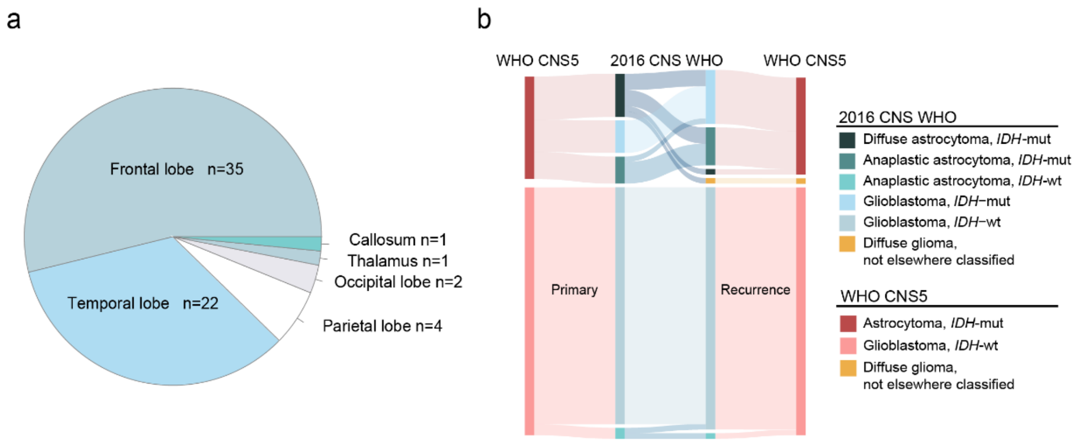

Supplementary Figure 1 Characteristics of the patient cohort

(a) Locations of all gliomas. Most of glioma were located at the frontal and temporal lobes. (b) Diagnosis classification based on 2016 CNS WHO and WHO CNS5. Diffuse glioma, not elsewhere classified indicated that the necessary diagnostic testing has been successfully performed but that the results do not readily allow for a diagnosis in WHO CNS5.

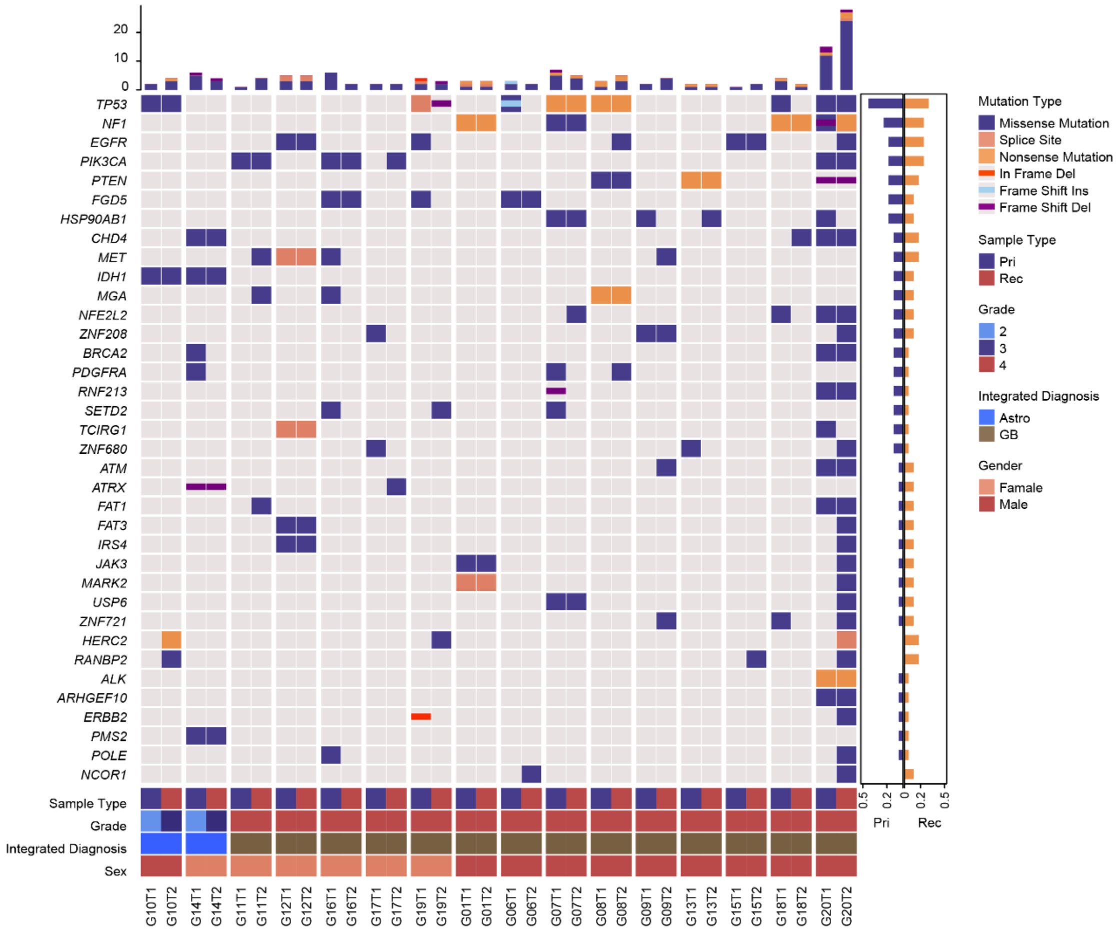

Supplementary Figure 2 Mutational landscape of gliomas and their matched recurrence (n=16). Mutation numbers, mutation profiles, sample type, grade, sex, CNS5 integrated diagnosis are shown from top to bottom of the panel. Recurrently mutated genes which occurred in at least three samples are showed on the left. The percentage of mutations in primary and recurrent tumors are depicted on the right of the panel.

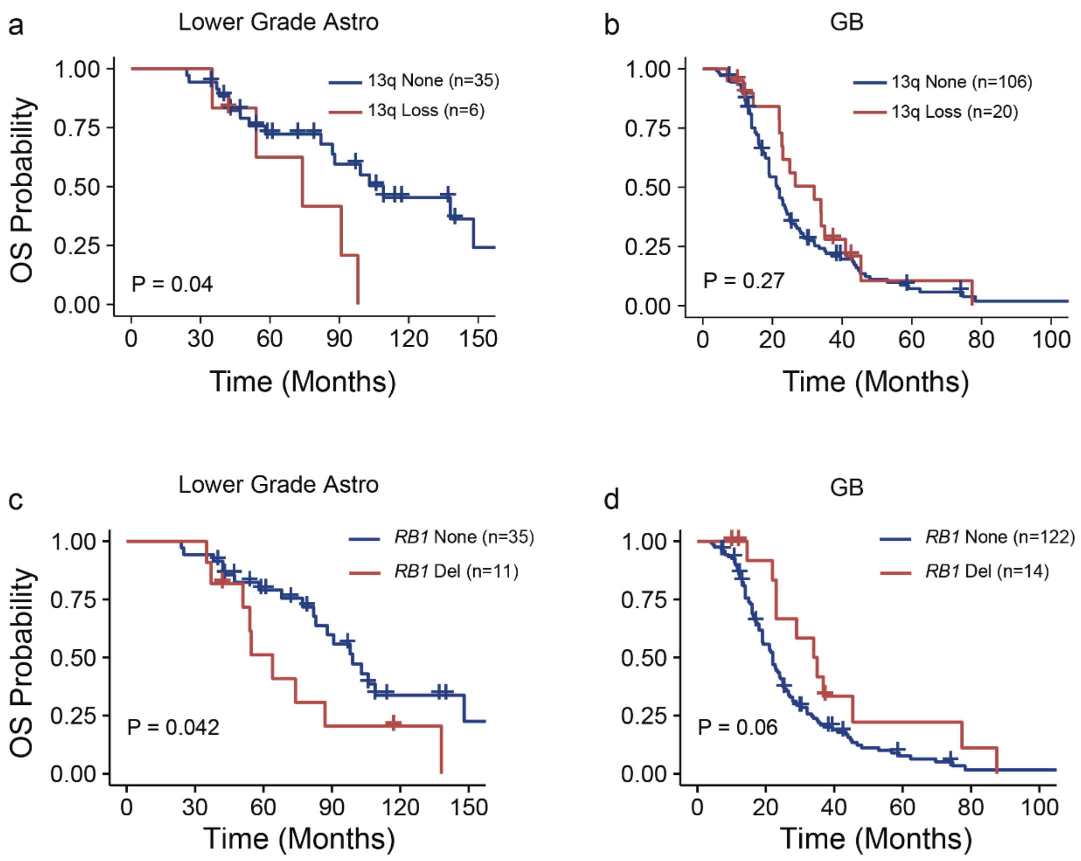

Supplementary Figure 3 Overall survival for lower grade astrocytoma and GB patients with or without newly acquired 13q loss and *RB1* deletion.

Overall survival for lower grade astrocytoma and glioblastoma patients with or without newly acquired 13q loss (a and b) and *RB1* deletion (c and d). P values were obtained from the log-rank test. Survival analysis was performed with the combination of our data (n=49) and public data downloaded from cBioPortal (n=187). Cases without 13q loss in both primary and recurrent tumors were classified into "13q None" group. Cases with 13q loss in recurrent tumor and without loss in primary tumor were classified into "13q Loss" group. Cases with *RB1* deletion in recurrent tumor and without deletion in primary tumor were classified into "*RB1* Del" group. Cases without *RB1* deletion in both primary and recurrent tumors were classified into "*RB1* None" group.

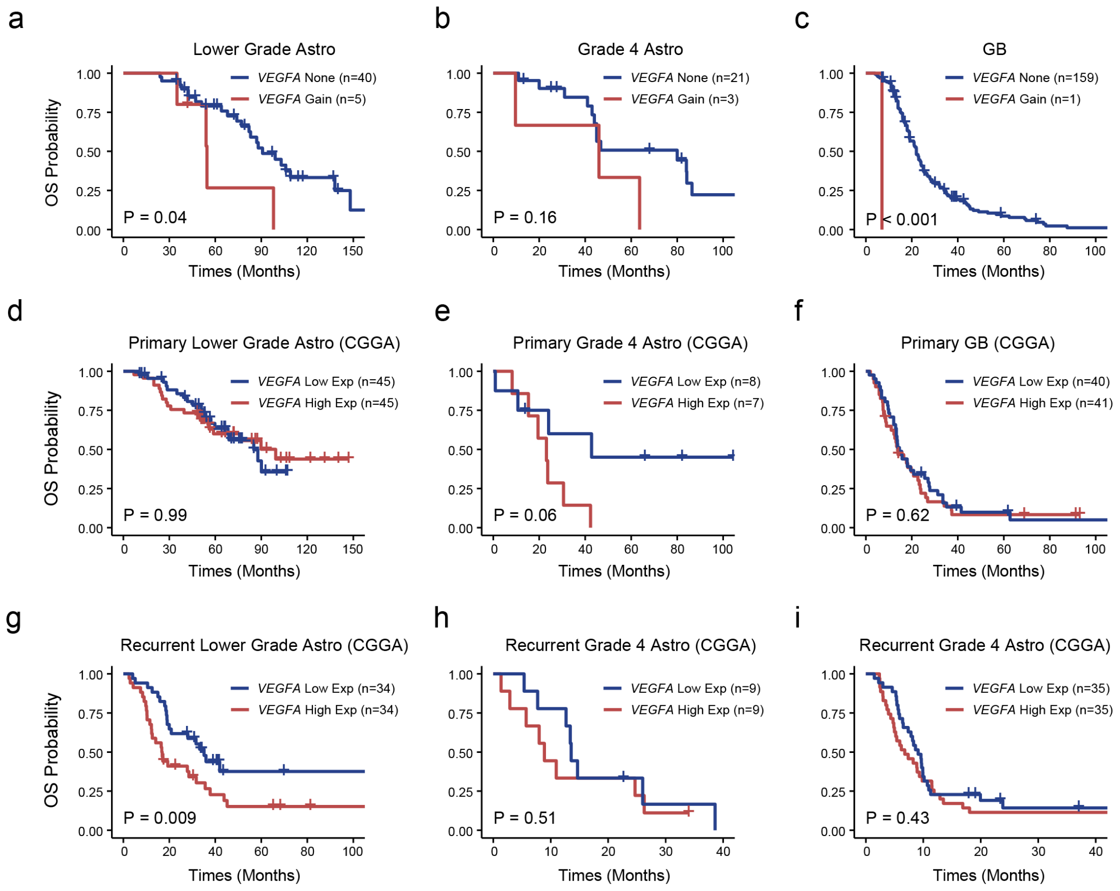

Supplementary Figure 4 *VEGFA* expression and prognostic value in lower grade astrocytoma, grade 4 astrocytoma and GB

(a-c) Overall survival between glioma patients with or without *VEGFA* gain, including lower grade astrocytoma (a), grade 4 astrocytoma (b), and GB (c). CNV data was from cBioPortal database. (d-i) Overall survival between glioma patients with high expression of *VEGFA* and low expression of *VEGFA*, primary lower grade astrocytoma (d), primary grade 4 astrocytoma (e), primary GB (f), recurrent lower grade astrocytoma (g), recurrent grade 4 astrocytoma (h) and recurrent GB (i). P values were obtained from the log-rank test. The expression data was downloaded from CGGA database.

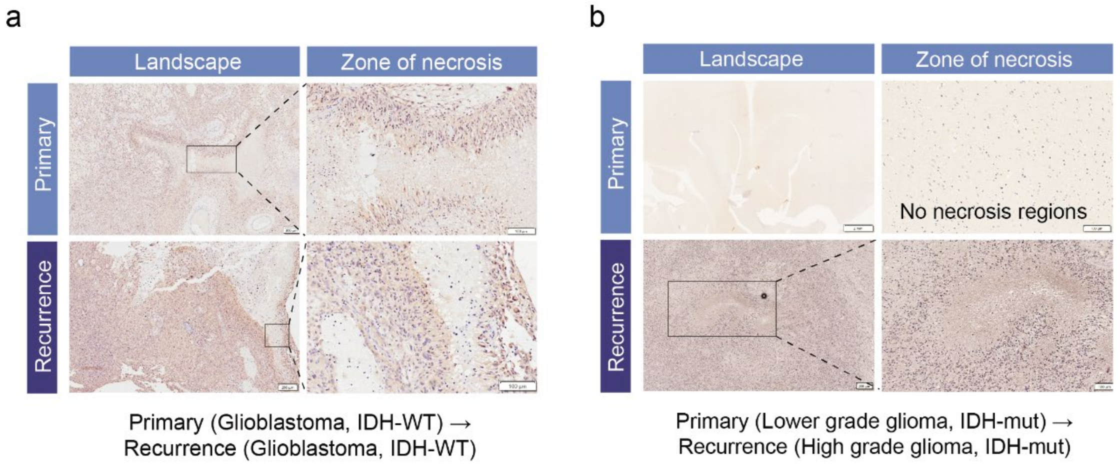

Supplementary Figure 5 *VEGFA* expression and prognostic value in lower grade astrocytoma, grade 4 astrocytoma and GB

IHC staining of *VEGFA* and regions of interest on paired primary and recurrent gliomas (a and b). The expression of *VEGFA* in necrosis regions in paired primary and recurrent gliomas. The presence of pseudo-palisade structures can be observed in both primary and recurrent GB (c) as well as the recurrent high-grade glioma (d).

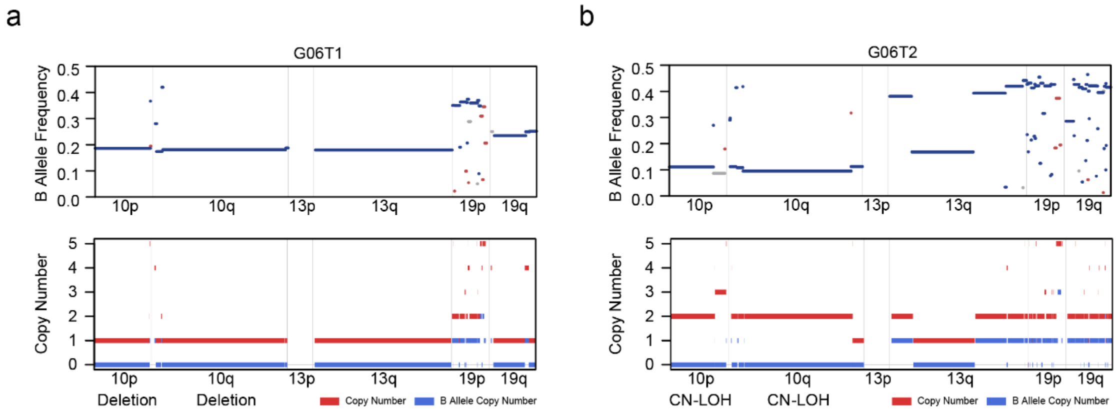

Supplementary Figure 6 Allele frequencies of SNPs and CNV profiles of G06. B allele frequency represents the lesser of the two allelic fractions as measured at germline heterozygous positions.
