# Supplementary material for: Distinct aneuploid evolution of astrocytoma and glioblastoma during recurrence
Source: NPJ Precis Oncol. 2023 Sep 23;7:97. doi: 10.1038/s41698-023-00453-1 (PMC10517995; doi:10.1038/s41698-023-00453-1)
Supplement: Supplementary file 2 — Reporting-summary [file 41698_2023_453_MOESM2_ESM.pdf]

nature portfolio

Corresponding author(s): Dandan Cao, Wei Hua, Ying Mao

Last updated by author(s): Jul 11, 2023

Reporting Summary

Nature Portfolio wishes to improve the reproducibility of the work that we publish. This form provides structure for consistency and transparency in reporting. For further information on Nature Portfolio policies, see our Editorial Policies and the Editorial Policy Checklist.

Statistics

For all statistical analyses, confirm that the following items are present in the figure legend, table legend, main text, or Methods section.

n/a	Confirmed	
☐	☒	The exact sample size (*n*) for each experimental group/condition, given as a discrete number and unit of measurement
☐	☒	A statement on whether measurements were taken from distinct samples or whether the same sample was measured repeatedly
☐	☒	The statistical test(s) used AND whether they are one- or two-sided<br>*Only common tests should be described solely by name; describe more complex techniques in the Methods section.*
☒	☐	A description of all covariates tested
☒	☐	A description of any assumptions or corrections, such as tests of normality and adjustment for multiple comparisons
☒	☐	A full description of the statistical parameters including central tendency (e.g. means) or other basic estimates (e.g. regression coefficient) AND variation (e.g. standard deviation) or associated estimates of uncertainty (e.g. confidence intervals)
☐	☒	For null hypothesis testing, the test statistic (e.g. *F*, *t*, *r*) with confidence intervals, effect sizes, degrees of freedom and *P* value noted<br>*Give P values as exact values whenever suitable.*
☒	☐	For Bayesian analysis, information on the choice of priors and Markov chain Monte Carlo settings
☒	☐	For hierarchical and complex designs, identification of the appropriate level for tests and full reporting of outcomes
☐	☒	Estimates of effect sizes (e.g. Cohen's *d*, Pearson's *r*), indicating how they were calculated

Our web collection on statistics for biologists contains articles on many of the points above.

Software and code

Policy information about availability of computer code

Data collection	No special software or code was used to collect the data.
Data analysis	Whole exome capture was performed using SureSelect Human All Exon V6 (Agilent Technologies). Adapters and low-quality reads were removed using trimmomatic (version 0.33). The clean reads were aligned to the human reference genome (hg19) with Burrows-Wheeler Aligner (BWA; version 0.7.10). All aligned reads were sorted, de-duplicated using Picard (version 1.103). Alignment quality assessment, local realignment and base quality recalibration were performed using the Genome Analysis Toolkit (GATK; version 3.1). Mapping rate, coverage rate and duplicate rate were calculated with qualimap (version .2.1). Genomic segments were identified using CNVkit (version 0.9.9). Somatic mutation calling was performed using the Mutect (version 1.1.4). Detected variants were annotated on the basis of Ensembl annotations (version 92) using Variant Effect Predictor (VEP; version 2.7). Oncogene were annotated using IntOGgen (https://www.intogen.org/). KEGG (Kyoto Encyclopedia of Genes and Genomes) pathways of the selected genes were enriched by KOBAS3.0 (http://kobas.cbi.pku.edu.cn/).All other statistical analyses were run using R (version 4.2.2).

For manuscripts utilizing custom algorithms or software that are central to the research but not yet described in published literature, software must be made available to editors and reviewers. We strongly encourage code deposition in a community repository (e.g. GitHub). See the Nature Portfolio guidelines for submitting code & software for further information.

Data

Policy information about availability of data

All manuscripts must include a data availability statement. This statement should provide the following information, where applicable:

- Accession codes, unique identifiers, or web links for publicly available datasets
- A description of any restrictions on data availability
- For clinical datasets or third party data, please ensure that the statement adheres to our policy

The raw sequence data reported in this paper have been deposited in the Genome Sequence Archive in National Genomics Data Center, China National Center for Bioinformation / Beijing Institute of Genomics, Chinese Academy of Sciences (GSA-Human: HRA004929) that are publicly accessible at https://ngdc.cncb.ac.cn/gsa-human. Public CNV profiles were downloaded from cBioPortal (https://www.cbioportal.org/). Public RNA-sequencing data (mRNAseq_693) was from CGGA (http://cgga.org.cn/index.jsp).

Research involving human participants, their data, or biological material

Policy information about studies with human participants or human data. See also policy information about sex, gender (identity/presentation), and sexual orientation and race, ethnicity and racism.

Reporting on sex and gender	Data regarding assigned sex of participants is listed in supplementary table 1.
Reporting on race, ethnicity, or other socially relevant groupings	There are no race, ethnicity or other socially relevant groupings.
Population characteristics	Provided in supplementary table 1.
Recruitment	This study is of a retrospective design. Tumor samples from cases were selected from the Biobank of Huashan Hospital. The Biobank collects tissue from neurosurgeries if the patient provides informed consent to participate in research. All samples are deidentified.
Ethics oversight	All studies were approved by the Ethical Committee and conducted according to the Helsinki Declaration.

Note that full information on the approval of the study protocol must also be provided in the manuscript.

Field-specific reporting

Please select the one below that is the best fit for your research. If you are not sure, read the appropriate sections before making your selection.

☒ Life sciences ☐ Behavioural & social sciences ☐ Ecological, evolutionary & environmental sciences

For a reference copy of the document with all sections, see nature.com/documents/nr-reporting-summary-flat.pdf

Life sciences study design

All studies must disclose on these points even when the disclosure is negative.

Sample size	Power analysis to determine sample size was not performed a priori.
Data exclusions	No data exclusion.
Replication	Our analysis integrated public data and our data, the results are reproducible.
Randomization	The retrospective design of this study does not necessitate randomization.
Blinding	The retrospective design of this study does not necessitate blinding.

Reporting for specific materials, systems and methods

We require information from authors about some types of materials, experimental systems and methods used in many studies. Here, indicate whether each material, system or method listed is relevant to your study. If you are not sure if a list item applies to your research, read the appropriate section before selecting a response.

Materials & experimental systems

n/a	Involved in the study
☐	☒ Antibodies
☒	☐ Eukaryotic cell lines
☒	☐ Palaeontology and archaeology
☒	☐ Animals and other organisms
☒	☐ Clinical data
☒	☐ Dual use research of concern
☒	☐ Plants

Methods

n/a	Involved in the study
☒	☐ ChIP-seq
☒	☐ Flow cytometry
☒	☐ MRI-based neuroimaging

Antibodies

Antibodies used	VEGFA Polyclonal antibody (1:200, Rabbit / IgG, Polyclonal, Proteintech, 19003-1-AP)
Validation	All antibodies were successfully validated using appropriate positive and negative controls.
